# Why can’t children with autism integrate into society in China? Study based on the perspective of NGO classification

**DOI:** 10.3389/fpubh.2023.1041815

**Published:** 2023-06-06

**Authors:** Zhiwei Li, Caiyun Qi

**Affiliations:** ^1^Department of Social Work and Social Management, Shandong Jianzhu University, Jinan, China; ^2^Department of Sociology, Shandong University, Jinan, China; ^3^Department of Social Work, Shandong University, Jinan, China

**Keywords:** children with autism, NGO classification, resource dependence theory, social integration, barriers

## Abstract

**Introduction:**

In the field of protecting children with autism, NGOs have become a major force that cannot be ignored. Although NGOs for children with autism have expanded the number and improved the quality of the services they provide, a large number of autistic children still cannot achieve the goal of social inclusion in China. The existing literature has mostly tried to explain the reason from the perspective of the common characteristics of NGOs and has paid insufficient attention to the huge differences between these NGOs, so it is impossible to identify the obstacles that children with autism encounter accurately.

**Methods:**

From the perspective of NGO classification, this study conducted an in-depth investigation of 4 NGO cases in City N, China, to show the impact of the difference of NGOs on the obstacles to the social inclusion of autistic children.

**Results:**

The research has found that under the authoritarian regime, NGOs for children with autism that rely heavily on external funds include three common groups: government-oriented NGOs, foundation-supported NGOs, and individual-financed NGOs. The structural characteristics of the funders and their interaction with the NGOs for children with autism shape their different action logics, as the result that the desire of children with autism to integrate into society cannot be achieved as expected.

**Discussion:**

The results of this study give more accurate insights into the barriers in social service provision for children with autism that impede their social inclusion and provide a reference for those seeking a solution to this problem.

## 1. Introduction

Autism spectrum disorder (ASD) has become a key public health concern facing all countries around the world. In China, the prevalence of ASD has increased, the current incidence rate being 1 in 100. Statistics show that there are over 10 million Chinese with ASD, more than two million of whom are children under the age of 12, and the number is growing by over 100,000 a year, making autism the fastest-growing developmental disability (1). Moreover, the treatment of autism over the lifespan is currently the most expensive medical expenditure payment (2); however, with early diagnosis and intervention, these costs can be reduced by two thirds (3). Therefore, early intervention therapies for children with autism are crucially important.

Socially-oriented intervention therapies such as community integration and the resumption of valued life roles are critical for the health and well-being of children with autism (4). According to the United Nations *Convention on the Rights of the Child*, children with autism in this study refer to people with autism under the age of 18 (5). This stage is critical for cognitive function, social interaction, and personality development (6). At this stage, improving the ability of autistic children to take care of themselves, adapt to society, and participate in social life can create opportunities for subsequent inclusive education, employment, etc., thus substantially changing their future life (7–11). *The International Classification of Functioning, Disability and Health (ICF)* model provides a clear, comprehensive and universal concept of disability, namely impairment of body functions and structures, bad activities and limitation of participation (12). Among them, social inclusion is an important part of participation, reflecting the extent to which individuals can participate in social activities. For children with autism, social inclusion contains at least two meanings: one is that children with autism acquire equal attention from the social, political, economic and cultural life; Second, children with autism are socially accepted and have relationships of mutual trust, appreciation and respect in the family and among friends and communities (13, 14). Historically, treatment methods for children with autism in the world underwent a transition from biomedical specialties and institutionalized care in a closed environment to training of social skills, collaborative learning group activities and community-based living in inclusive environments (15–17). In China, the government has also clearly expressed its commitment to the full inclusion of children with autism into society. State laws and regulations, such as *the Law of the People’s Republic of China on the Protection of Persons with Disabilities* (1990, 2008), *the Regulations of the People’s Republic of China on the Education of Persons with Disabilities* (1994, 2017), and *the State Council on the Establishment of a Rehabilitation Assistance System for Children with Disabilities* (2018) all pay special attention to autism groups. Furthermore, programs such as “Colorful Dream” and “Learning in Regular Class” are also committed to providing integrated services for children with autism – China is establishing a rights-based social service system for autistic children with the goal of social inclusion (18).

The role of non-governmental organizations (NGOs) in providing social inclusion services to children with autism cannot be ignored. NGO is a form of organization which is independent of the government system, with non-profit, voluntary, organizational, autonomous, private and other attributes (19). In Western Europe, behind the state-centered “welfare state” is the supply of social welfare through the reality of government-NGOs partnership (20). Since the collapse of the Soviet Union, two parallel developments have also taken place in former Communist countries, namely, the systematic withdrawal of the state from the provision of social services and the increasing provision by NGOs in various fields (21). In the field of social services for autistic children, NGOs have been established to address government and market failures. For a long time, governments and markets have been criticized for their slow response and their lack of vision on rights, respectively (22–24). In China, the first NGOs for children with autism developed in the 1990s with the goal to speak for children with autism and their families and fill the gap in social welfare services from the nation and the market (25, 26). Encouraged by the Chinese authorities, NGOs have shown great development potential (27). Data from *Report on the Development of Autism Education and Rehabilitation Industry in China III* shows, from 2016 to 2019, the number of NGOs for autism increased by 12% from 1,600 to 1811, and service capacity increased by 30% from less than 200,000 to more than 300,000 (28). With the promulgation of the *Government Procurement Law of the People’s Republic of China* (2014), it is increasingly common for governments to purchase social services from NGOs. NGOs have become the most important providers of social inclusion services for children with autism. Although all of the efforts of NGOs are for the well-intended purpose of giving children with autism equal rights, dignity, and respect, unfortunately, we were surprised to find, through long-term research, that the social inclusion aims undertaken by NGOs cannot be fulfilled.

Why are children with autism unable to achieve the goal of social inclusion through NGOs? There are a few direct and systematic responses to this question, and these researches start from one of three angles. First, the government’s policy has produced the exclusion results. On one hand, most scholars believe that in countries represented by China, NGOs for children with autism are heavily controlled by the government (27), and official policies are mainly focused on children aged 0 to 6, thus leading to designated NGOs preferring to provide services for younger children instead of older children with autism (29). On the other hand, compared with Western Europe and North America, NGOs in developing countries are currently giving priority to service provision rather than to advocacy or the implementation of disability rights (30). Due to long-standing social barriers for people with disabilities, most NGOs assume that the cost of inclusive services is too high to justify; therefore, it is impossible to elicit sympathy to gain funding from the government (31, 32). Second, lack of money and human capital leads to poor service provision, especially in the case of NGOs that get no government support. Because of their unofficial status, the professional capacity of practitioners working in NGOs for children with autism is not certified and their pay is relatively low, resulting in a large loss of talent and thus affecting the effectiveness of services (33). In addition, in the poor charity environment in China, NGOs for children with autism can hardly acquire stable and institutionalized social funds to help these children (33). Third, the uniqueness of social inclusion service projects makes it difficult to implement. Compared with other interventions, social inclusion services often require cross-agency cooperation. However, evidence shows that different agencies are in a gaming environment, and the inconsistent goals make it difficult to implement and maintain cross-agency cooperation in social inclusion projects, which is prone to failure (34–36).

We do not deny the reasons proposed in the above literature, but our in-depth qualitative research indicates that the reality does not stop there. Existing studies, on the one hand, mostly explain the reason from the government-NGOs relationship approach, ignoring the perspective of NGO classification and the diversity of constraints based on this. That is, the current literature mostly explains from a single (category) case in an attempt to summarize the common characteristics of NGOs instead of the huge differences between them. In fact, various NGOs have different action logics. If we generalize NGOs, then it is impossible to accurately locate the underlying dynamic mechanism that explains why children with autism encounter obstacles to social inclusion. On the other hand, the current literature has discussed NGOs for autistic children in developed and developing countries, but has paid insufficient attention to this issue under the authoritarian regime, thus unable to reveal a variety of forms of control on NGOs for autistic children in China, and the exclusive behavior it produces.

Therefore, this study tries to explore the reasons why social inclusion is not achieved for children with autism as expected in the context of China from the NGO classification approach. We first explain the role, basic functions, and development difficulties (mainly in terms of funding) of Chinese NGOs for children with autism and then classify these NGOs according to their funding sources. After that, using case study method, four NGOs for children with autism in City N in China are investigated to examine the different action logics and consequences in terms of NGOs providing social inclusion for children with autism. This study is critically important to promoting the practice of public health services for children with autism and will effectively fill a gap in the current literature.

## 2. NGOs for children with autism in China and their classification

In 1993, the first autism educational organization was established by the mother of a child with autism simply because her child could not secure an educational placement in public schools (37). Since then, parents of children with autism and people with passion and charity have set up more and more NGOs to serve children with autism and their families (25, 38). In recent years, the Chinese government has relaxed requirements for a dual management system^1^ and has been increasingly outsourcing social services (39), which has further stimulated the explosive growth of NGOs for children with autism. What’s more, the number and proportion of social inclusion services for autistic children, such as integrated education, community rehabilitation, employment support, recreational and sports activities, and peer activities, are also increasing. Research shows that NGOs for children with autism are playing an increasingly important role in mobilizing resources, providing public services, and promoting social participation and inclusion for children with autism (40).

NGOs receive funding from various sources. Funders include the government, as well as other sources, such as social fundraising, sponsorship, and donations. At the same time, NGOs may obtain funding through market activity income (41). In addition, international assistance is also an important source of funding for NGOs in developing and underdeveloped countries (42, 43). According to statistics, in 2019/20, the largest source of income for NGOs in the UK was the public, including donations, legacies, charity stores, and membership fees, accounting for 51% of all income. Government support accounts for 26%, including grants and public service contracts. Other sources include the voluntary sector (9%), investment (9%), the private sector (4%), and national lottery (1%) (44). The Chinese people lack enthusiasm for the investment of NGOs, and the government is their main donor. According to data, nearly 50% of the funding of NGOs comes from financial allocations, 21.18% comes from membership fees, 6% comes from market income, 5.63% comes from sponsorship and project funds provided by enterprises, and 5% comes from other sources (45). Currently, compared to other countries, there are three trends in the funding of NGOs in China: (1) The Chinese government is increasingly using tangible support tools such as purchasing service contracts, tax preferences, and subsidies, among other means, to incentivize NGOs; (2) Private foundations have developed rapidly and have become increasingly active in supporting the development of grassroots NGOs; (3) Foreign funds are becoming increasingly unavailable. In particular, the implementation of the *Administration of Domestic Activities of Overseas Non-governmental Organizations* (2016) has led to NGOs for children with autism facing increasing challenges in attaining overseas funds, and the *Charity Law* (2016) has further restricted the channels for public foundations to engage in raising social funds in China (46, 47).

According to resource dependence theory, access to resources in such situations is crucial. Resource dependence theory is an important organization theory that studies the relationship between organizations and external resources and the environment, and its basic assumptions are as follows: (1) survival is what organizations focus most on; (2) for survival, organizations need to obtain external resources because no organization can be fully self-sufficient; (3) thus, organizations must interact with elements from the environment on which they depend, and these elements are often contained in other organizations; (4) the survival of an organization is based on its ability to control relationships with other organizations (48). NGOs must acquire the resources needed for survival, which can be achieved in the form of exchange, trading, or donation. In most cases, however, this is an asymmetric dependency relationship: The resources that the recipient needs to maintain its survival are in the hands of the sponsor, so the recipient must meet the requirements of the sponsor in order to obtain the resources, whereas the sponsor has the absolute power to reshape or constrain the recipient’s behavior because of its unique advantages in terms of resources (49, 50).

Through literature review and field research, it is found that NGOs for children with autism rely most heavily and critically on the outside world for funds, which come from government, foundations, market income, donations from enterprises and individuals, self-financing by parents of autistic children, and so on^2^. Due to different funding sources and ways to serve sponsors, NGOs for children with autism have developed different models. Firstly, to promote social welfare, different government departments will provide different types of government assistance to NGOs (51). Among them, the government purchasing of services^3^ “has become the most attractive tool” (52). Secondly, fundraising, sponsorship, and donations from the society also play an important role (53). In order to address the growing social demand and inequality, the government has encouraged the rapid growth and expansion of foundations by introducing of the *Foundation Management Regulations* (2004) and the *Charity Law* (2016). Foundations have become one of the important sources of funding for NGOs (47, 54). The development of philanthropy has also stimulated the enthusiasm of some enterprises and individuals to donate money (55, 56). However, the object is selective, whether it is the government or social support. The two donor types have jointly created a result-driven and institutionalized environment in which NGOs easily lose sight of their missions and uniqueness (1), leading to an inability to meet the needs of autistic children and their parents. In the face of this situation, some parents of autistic children have voluntarily raised funds to establish grassroots NGOs to serve autistic children, to truly and effectively respond to their needs. In addition, market income is also one of the funding sources for NGOs for children with autism. However, families with disabled children generally have poor economic conditions and low effective market demand (57), so it often exists as supplementary funds for NGOs for autistic children. Based on this, we have selected three typical and common modes from these different types: government-oriented, foundation-supported, and individual-financed NGOs. What must be stressed is that we construct these ideal types in a taxonomic sense and try to explain their inherent operating mechanisms. However, in field research, the characteristics of NGOs are more complex: NGOs have the main characteristics of a certain ideal type but concurrently contain components of other types.

### 2.1. Government-oriented NGOs for children with autism

Government support generally includes two ways: government subsidies and government purchase of services. Among them, the government purchase of services, as a key measure of the government to promote the development of NGOs (58), plays a more important role. Most of these NGOs are designated units of government purchase services and have diverse and formal contractual relationships with the government. To gain support, NGOs’ activities often focus on the government’s priorities. In 2018, with the promulgation of *the State Council on the Establishment of a Rehabilitation Assistance System for Disabled Children (2018)*, local governments made great efforts to purchase rehabilitation services for disabled children, and data showed that more and more NGOs for autistic children had become designated units of the government purchase of services.

### 2.2. Foundation-supported NGOs for children with autism

Foundations, with their unique social mission, value proposition, and resource advantages, have been called a blessing for grassroots NGOs. In terms of the supply of funds, foundations provide NGOs with resources through project bidding or service purchases, and NGOs for autistic children also actively seek funds from foundations to meet their own needs. With China’s support for the foundations, more and more foundations have emerged, such as the China Poverty Alleviation Foundation, the Nandu Public Welfare Foundation, the Tencent Foundation, the One Foundation, the Huiling Foundation for the mentally disabled, and so on. Such NGOs are deeply influenced by the principles of the foundations, and their services are also carried out in accordance with them.

### 2.3. Individual-financed NGOs for children with autism

These NGOs are mostly spontaneously established by parents to cope with the current lack of policy and professional support. In the survey, it is found that the parents of autistic children are more likely to gather together, and to spontaneously and actively establish various social groups and organize various activities to promote social inclusion to meet their children’s needs. This is less common in other types of parents with disabilities. This is related to the rapid growth of autistic children in recent years, and the current social service support system cannot meet the needs of autistic children. They usually receive funds from parents, volunteers, or private donations for autistic children. Most of these organizations are relatively small and unregistered, aiming to carry out activities that mainstream NGOs cannot.

## 3. Method

This study adopted case study method to explore the differences in terms of the achievement of social inclusion for children with autism between NGOs supported by different sources of funds and the reasons for these differences. The detailed, nuanced case study approach, which focuses on the interdependencies of the various parts of an event and the ways in which these relationships occur, is well suited to answering questions left in the black box about how NGOs respond to different environmental pressures (59, 60).

### 3.1. Field access and sampling

We selected City N as the main research site. City N, the provincial capital of Province J, is situated in northeastern part of China. In 2014, there were over 20,000 children with autism in City N, with an incidence rate of 1 in 68. This figure is very high among Chinese cities, and the trend of increase is obvious. Therefore, the problem of autistic children has attracted considerable attention from the government, charitable foundations, and all sectors of society in City N. At present, City N is one of the places in China where NGOs for children with autism are developing quickly and are relatively mature. In terms of number and type, there are many NGOs for autistic children, and the difficulties NGOs encounter are very typical.

The field work for the study began in February 2021. With the help of the City N’s Disabled Persons Federation (DPF)^4^ and the recommendation of NGOs for autistic children and parents, the authors conducted an extensive preliminary survey of various NGOs for children with autism in City N, classified the NGOs, and defined their typical characteristics. In order to ensure the category representativeness of the sample (61) and explore the significant differences between different fund-dependent NGOs, purposeful sampling was used in this study following three standards: the case (1) has typical characteristics of the NGOs for autistic children classified in this paper, that is relying on government funding (government-oriented NGOs), relying on foundation funding (foundation-supported NGOs), or mainly relying on self-raised funds (individual-funded NGOs); (2) has established for more than 3 years and have rich work experience serving children with autism (such NGOs can provide more information); and (3) has a relatively stable source of funds (in order to control the influence of capital changes and other factors on the results). Finally, we selected four NGOs for comprehensive observation and in-depth interviews: AH Rehabilitation Training School for Special Children (AH), a government-oriented NGO; BI Training Center for Special Children (BI), a foundation-supported NGO; CJ Social Work Service Center for Families with Mental Disabilities (CJ), an individual-financed NGO; and DK Services Center for Families with Mental Disorders (DK), an individual-financed NGO. In the investigation, we found that there were obvious differences in individual-financed NGOs due to the different composition of parents. To fully illustrate the complex characteristics of NGOs, we chose two cases^5^. Sample details are shown in Table 1.

**Table 1:** Cases of NGOs for children with autism.

Type	Government-oriented NGOs for children with autism	Foundation-supported NGOs for children with autism	Individual-financed NGOs for children with autism	
			Elite-funding-oriented NGOs for children with autism	Small-scale crowdfunding-based NGOs for children with autism
Name	AH	BI	CJ	DK
Date of establishment	2010	2003	2016	2015
Number of autistic children	Above 200	28	Above 200	Above 80
Work sites	A fixed place with full facilities	A fixed place with full facilities	Working in community	None
Service content	Exercise training, homework training, speech training, sensory training, physical therapy, music therapy, game class, etc.	Community living, evening family accommodation, pre-employment training, assisted employment, etc.	Community living, outdoor activities and socializing, integrated employment, skills training, etc.	Community living, social inclusion activities, art therapy, parent training, etc.

### 3.2. Data collection and data analysis

With the assistance of the heads of the NGOs for children with autism, the authors collected ethnographic data from March to July 2021. Three different methods were adopted: participatory observation, in-depth interviews, and access to textual materials such as government planning outlines, statistical yearbooks, archives of NGOs, and other files. Ethical approval for this study was obtained from the Academic Committee of School of Philosophy and Sociology, Jilin University.

First, the first author conducted intensive participatory observations of each NGO, respectively, for 15 to 20 days in order to take a closer look at the day-to-day work of these NGOs, especially social inclusion service project application, social inclusion service supply, social inclusion service project assessment^6^. At the same time, the ethnographic field work also included participation in characteristic courses and social activities related to social inclusion, such as hiking, mountain climbing, film watching and so on. During that time, the staffs and parents generously shared their work experiences, feelings, and attitudes toward the social inclusion of children with autism with the researchers. These conversations were recorded as an important raw data source.

Second, we conducted 33 semi-structured one-to-one and one-to-many interviews (including 5 follow-up interviews). The interviewees included 21 NGO staff members (including 5 in AH, 6 in BI, 5 in CJ, and 5 in DK) and 16 parents of autistic children. In accordance with the principle of purposeful sampling, the inclusion criteria for interviews with NGO staff are: (1) the founders and principal managers of NGOs; (2) rehabilitators, special education teachers or social workers with more than 3 years of working experience and rich experience in social inclusion services; (3) Consent to attend the interview. The inclusion criteria for parent respondents were: (1) the primary caregivers of autistic children; (2) Their children are aged 0–17 years old; (3) Receiving/having received social inclusion services at the four NGOs; (4) Enough Chinese fluency to participate in the interview process. In order to improve the reliability and validity, we fully consider the heterogeneous factors such as age, gender, education, occupation, position of NGOs staff interviewees, and also fully takes into account of variables control to family income, *hukou*, education level, age, gender, disability grade, disability type of the children with autism of parents’ interviewees. The data collection process was achieved through semi-structured interviews, in which the NGO staff respondents were asked to answer questions about the NGO’s funding sources, working modes, types of social inclusion service supply, coverage, and work barriers. Parents were asked to answer questions about their access to social inclusion services for autistic children, their experience of exclusion in social inclusion services, and their attitudes toward different NGOs. Before the interviews, participants were provided with information about the purpose of the study, the survey schedule, the duration of the study, and their rights and risks. The researchers conducted all of the interviews in Mandarin, transcribing the interviews verbatim into Chinese and selectively translating them into English as needed to present the findings. The whole research process strictly followed the requirements with regard to participant confidentiality, no harm, and informed consent.

Third, with the consent of the NGOs’ leaders, we read some of their internal files. At the same time, we collected planning outlines and policy documents related to rehabilitation services for children with autism in City N to supplement the field data.

We integrated and analyzed data from different sources to form a consistent theme and reveal why children with autism are facing social inclusion difficulties in different categories of NGOs for children with autism. Our aims were to develop an extended case study of both theoretical and practical significance (62), to have a dialogue with the existing literature on the social inclusion dilemma for children with autism, and to offer certain recommendations on future rehabilitation policy practices for children with autism. Meanwhile, following the triangle method (60), the research team applied as many research techniques as possible, obtained information and materials from various data sources around the research topic, and compared the consistency and difference between the results to make the research conclusions more credible and accurate.

## 4. Findings

In government-oriented, foundation-supported, and individual-financing NGOs for children with autism, the structural characteristics of the funders and their interactions with the recipients shape NGOs’ different action logics. Under the tension of the efficiency mechanism and rights protection mechanism dominated by NGOs’ need for survival, there are various obstacles to the supply of services, such as group selection and service replacement, and consequently the aspiration to integrate children with autism into society cannot be achieved.

### 4.1. Government-oriented NGOs for children with autism

Government-oriented NGOs for children with autism establish close ties with the government mainly through the government’s service purchasing system. Since 2009, the government has allocated financial expenditure to fund the Salvage Rehabilitation Project for Poor Children with Disabilities, and in 2018, it officially established a Rehabilitation Assistance System for Children with Disabilities, allocating special subsidies for rehabilitation training for children with disabilities, including autism. During this period, a large number of NGOs, such as AH, grasped the policy opportunities, becoming designated units for the purchase of rehabilitation services and experiencing rapid development. However, this approach is not always helpful. Influenced by the institutional heritage of omnipotent government, administrative power has long been centralized in China, while social forces are far from mature (63). The government decides the content and objectives of services, has complete bargaining power, and designs complex standards for strong supervision and strict assessment. As a result, under the institutional environment in China, the equal contractual relationship contained in the service purchasing system is in practice implemented on the basis of the principle “I (the government) pay, and you (the NGO) do the work” (64). Our research found that this unequal government-NGO relationship continues to produce and reproduce barriers that prevent children with autism from achieving social inclusion.

#### 4.1.1. Age exclusion

NGOs for children with autism provide rehabilitation services for younger children with autism, thus the rights of older children with autism cannot be guaranteed. Under the authoritarian system, local DPF maintained the hierarchical relationship through bureaucratic management and strong assessment and supervision, rather than the original contractual relationship. In order to get funding, AH has had to become the “steward” of the local PDF, and thus it tends to implement the PDF’s will. Lili, AH’s Teaching Director, stated: “*At present, it is not up to us to decide. It depends on which areas the local DPF will invest more money in, and our focus will shift accordingly” (Interview with Lili, March 10, 2021)*. The rehabilitation training subsidy in City N is only provided to children with autism aged 0 to 6 years, and therefore most government-oriented NGOs have turned to providing social services for younger children with autism, so there is an obvious age exclusion. Many parents of children with autism commented on the lack of services for children over the age of seven (field notes, May 2, 2021). What is worse, under this top-down institutional structure, there is no space for bottom-up service users to exercise autonomy, and they cannot effectively fight for the rights of older children with autism.

#### 4.1.2. Service type exclusion

The unequal bargaining power between funders and service providers limits the delivery of social inclusion courses. Here, unequal bargaining power means that the price is not regulated by the market but rather set solely by the government (64). In the local DPF’s curriculum directory for NGOs, we clearly found the names of services and their prices: for example, “speech training, 30 yuan per class; sensory inclusion training, 40 yuan per class” (provided by the AH Archives, March 18, 2021). Because NGOs for children with autism are responsible for their own profits and losses, they prefer to provide services that are highly profitable. Unfortunately, social inclusion courses for children with autism only appear sporadically in the curriculum directory, and the prices for such courses are relatively low, which discouraged Ming, the principal of AH,

Social inclusion is easier to say than to do. It does not mean taking them to normal society to play or letting them communicate with normal children. If we want to do good social inclusion, we must recruit workers with appropriate skills, find venues, and reset the training plan. Now with these little funds, we cannot do it. (Interview with Ming, March 13, 2021)

#### 4.1.3. Reduced service quality

In order to ensure the effective implementation of tasks, the government has a strict, complex, and cumbersome assessment system. AH has incurred considerable management costs to comply with these systems and has correspondingly squeezed its investment in the service it provides. The assessment content of local DPF includes site setting, facilities and equipment, human resources, management system, quality control, etc., and the assessment means include data access, field observation, service effect evaluation, questionnaire survey, etc. AH therefore invested a lot of time and energy to meet the site settings, facilities and equipment, human resources, management system requirements, while ignoring the input of social inclusion services. What’s worse, In China, the fund for each child with autism is provided through the local DPF in the place where the child’s *hukou* is registered, and the criteria for receiving the fund vary from region to region. At a peak, AH will deal with the assessment and audit requirements of nearly 20 DPFs, and the labor cost of processing these materials will inevitably rise. Lili complained that *“the tasks that one staff member could accomplish in the past are now simply impossible” (Interview with Lili, March 10, 2021)*. Another issue is that the staff turnover rate is very high in NGOs for children with autism (33). The downward pressure of assessment makes the work team, which is already short of personnel, unable to focus on rehabilitation services; and this can be regarded as depriving children with autism of their rights.

#### 4.1.4. Service content replacement

The assessment system also leads to a bias in course selection. AH is forced to replace those services that are not conducive to the assessment, and social inclusion services bear the brunt of this process. Actually, the varied assessment criteria of DPFs provide AH workers with their worst nightmare: they must comply with punitive rules to satisfy their sponsors. But these punitive rules do not always adapt to realities. In the face of the assessment dilemma, NGOs for children with autism often overlook, intentionally or unintentionally, some of the service needs that are not conducive to the assessment. High-demand social inclusion services have been replaced by mainstream services, such as ABA, because of their excessive complexity and uncontrollability. If they cannot replace courses, NGOs turn to changing the detailed content of courses. In one interview, Jun, a therapist, recalled a time when they “faked” classes,

The DPF required us to have no fewer than four classes a day, but the teachers could not arrange them, so we set up an extracurricular activity class as a social inclusion class to solve the assessment. (Interview with Jun, March 17, 2021)

Not surprisingly, AH is often criticized by other NGOs for children with autism as being “utilitarian” and “indifferent.” AH has to report annually to the local DPF on its customers’ welfare qualifications and service use, and it obtains survival resources by relying on the government, which is an inevitable choice for its development. But in this process, the social inclusion progress of children with autism is hindered.

### 4.2. Foundation-supported NGOs for children with autism

The funding provided by foundations that support NGOs for children with autism has complementary advantages for both sides, each taking what it needs (65). Although presenting the appearance of friendly cooperation, the rational logic of the pursuit of profit maximization embedded in the power game leads to an unequal relationship between the two sides, where rich foundations with overwhelming power dominate the practices of NGOs for children with autism (66). The most important manifestation of this is project competition.

Projects are the life of foundation-supported NGOs, and their capacity to apply for projects directly determines their survival and development. Jiang, the principal of BI, showed that from foreign foundations to domestic foundations and then to charitable organizations on the Internet, the sources of funding have changed a lot. However, there has been no change in the method of open bidding for projects (Interview with BI President Jiang, April 4, 2021). In recent years, with policy restrictions, funding constraints, and the transformation of foundations, project applications seem to be getting harder and harder and competition is getting keener. In order to choose the final funding target, a foundation fully considers every project’s advantages and risks, mainly referring to three standards: impact, implementation difficulty, and NGO’s working experience (66). The strong competition has made it a priority for NGOs to meet the selection requirements of the foundations instead of the needs of children with autism. As a result, a large number of children with autism are excluded from the service system.

#### 4.2.1. Impact rather than quality

Initially, foundations equate the impact of projects and NGOs with quality of service. However, in the process of making the impact quantitative, impact gradually becomes a rigid digital competition, losing the meaning of “quality.” BI, originally established with the support of a caring person from Hong Kong, with most of its funds initially coming from foundations in Hong Kong. In recent years, they have begun to seek support from domestic foundations due to restrictions on overseas funds.

Because of political security, it is difficult for foreign foundations to support us now. In the past two years, we stopped an important project supported by the Hong Kong Foundation. Currently, we mainly apply for funds from domestic foundations, some famous ones, such as Tencent Charity Foundation, One Foundation, etc. Nowadays, there are too many NGOs applying, and the competition in obtaining funds is becoming increasingly fierce. From the perspective of the foundation, they must also want to support those with strength and great social attention that can bring flow or help to the development of the foundation. (Interview with Jiang, April 4, 2021)

But they are often in a “helpless” state because of digital competition,

On the Tencent 99 Charity Day^7^, labor and wealth are wasted, completely losing their original intention. Now, to obtain funds, one has to donate steps and draw small red flowers. This consumes too much energy. If it were not for working with the ** Foundation and making participation mandatory, we would not have wanted to do it.. (Interview with Jiang, April 4, 2021)

Moreover, in order to expand its impact and improve the success rate of social inclusion programs, BI gives more attention to cultivating successful cases than to bringing universal benefits to children with autism. Koji, the program director at BI, is particularly focused on high-functioning children with autism because *“once their potential is played out, they will be the best advertisement for the institution” (Interview with Koji, April 6, 2021)*. However, the model of infinitely increasing the success of a small proportion of children with autism may bring about the concentration of resources and new forms of unfair resource distribution.

I think the most serious problem is that the public has misconceptions about autistic children. They think autistic children will be gifted in some way, such as playing the piano, or drawing... However, very few autistic children are gifted. Most of them, like my child, cannot do anything, and it’s difficult for them to take care of themselves. This misunderstanding is because these NGOs always favor and promote that type of children. The real-life of autistic children is not seen. (Interview with Pei, the parent of an autistic child, April 13, 2021)

#### 4.2.2. Mild autism rather than severe autism

Faced with competition and assessment pressure from different foundations, foundation-supported NGOs for children with autism tend to select children with mild symptoms who are most likely to meet all the assessment criteria from foundations. For example, an integrated employment project for youths with autism is BI’s most competitive project. In order to maximize funding, BI makes this project meet as many foundation standards as possible. However, different foundations have different requirements. In this case, foundation-supported NGOs for children with autism select children with a low degree of disability as it enables them to meet all requirements. While this is the best choice for BI, it results in the relentless rejection of a large number of children with severe autism.

Children with mild autism are easier to manage and have better employment inclusion effects. Children with severe autism are difficult to manage and can hit you easily. Now, these children are so tall and powerful that you cannot control them. It’s very difficult for us to do social inclusion services, and it’s likely that we will not be able to do anything. (Interview with Koji, April 6, 2021)

#### 4.2.3. A few rather than all

The competition mode, also known as the “survival of the fittest” mode, will eliminate many of the less developed NGOs and the children with autism they serve. NGOs that have already worked with foundations are well positioned as their capacity to provide services is recognized and there is an established trust mechanism and communication method between them and the foundations, all of which is extremely important for future cooperation. However, there is a paradox in this: The weaker NGOs in need of foundation support are more likely to fail to get support due to lack of ability, and this deprives the children with autism served by these NGOs of their right to receive social inclusion services. It means that weak foundation-supported NGOs for children with autism lack opportunities to improve their capabilities and also reduces the possibility of them obtaining resources, thus further limiting their survival and development and inevitably stifling their vitality and creativity.

The current situation is “the strong stronger, the weak weaker.” Only when you develop and achieve something will the foundation be willing to invest in you. (Interview with Shui, April 11, 2021)

### 4.3. Individual-financed NGOs for children with autism

Individual-financed NGOs for children with autism are often set up spontaneously by parents with autistic children, rooted in the community, and financed through personal donations. These NGOs have emerged to express dissatisfaction with bureaucrats and experts (67). The birth of an autistic child often makes their parents second-class citizens, and children with autism’s needs in terms of equal participation and social inclusion do not provoke a positive response from bureaucrats and experts, whose ignorant and indifferent attitude makes parents feel helpless (67). Parent-support organizations, on the other hand, provide an integrated environment in which children with autism and their parents can feel relaxed, which is more helpful for them (68).

Our research found that, unlike the other two types of NGOs, in individual-financed NGOs for children with autism, the funds basically come from parents and social donations and are jointly managed by the members. Generally, the core members who contribute the most decide on course themes and activity plans on the basis of their own needs and preferences. Due to the different economic and social backgrounds of the members and the different amounts of resources they have obtained, there are different individual-financed NGOs, the common ones being elite-funding-oriented NGOs and small-scale crowdfunding-based NGOs. The results of the field research showed that these two types of NGOs are unable to achieve good social inclusion for children with autism, but the specific reasons for this are clearly different.

#### 4.3.1. Elite-funding-oriented NGOs for children with autism: multiple exclusions under elite power

The core members of elite-funding-oriented NGOs are mostly elite families. In CJ, the core members are mostly university professors, doctors, lawyers, and successful businessmen. They surpass most people in ability, insight, courage, property, cultural literacy, and many other aspects. For the rehabilitation of children with autism, they use their own money, knowledge, and social capital to establish mutual-aid organizations for parents and their autistic children and successfully attract other elite parents. They also make full use of their professional ability to undertake social inclusion projects such as “Light up the star,” “*Yiqi* Travel,” and “Friendly campus tour,” which have become extremely popular across China. However, the strong personal characteristics and power advantages of elite groups inevitably produce social exclusion behaviors in the construction of inclusive social environments.

##### 4.3.1.1. Interest priority

Elite-funding-oriented NGOs get a lot of attention and funding through personal networks or organizational influence through many enterprises. In order to protect the maximized profit of individuals and these enterprises, elite members often undertake some semi-inclusion or even non-inclusion activities. This approach is well suited to successful collaboration, but it does not fully achieve social inclusion. For example, Ken, the parent of an autistic child, made the following comments about movie watching activities,

At CJ, communication activities will be organized between healthy children and parents, and autistic children like us. However, at this time, I feel we are like animals in zoos, for people from outside to visit and sympathize. (Interview with Ken, April 29, 2021)

This is far from what parents expect. What is more difficult for sensitive parents of autistic children to accept is that every time in such activities, they have to stand in a row, accept donations from kind-hearted individuals and enterprises, and force autistic children and their parents to take photos with some banners. As Ken said,

This is using the kids totally as a promotional tool for profit. (Interview with Ken, April 29, 2021)

##### 4.3.1.2. Elite capture

Although CJ says that it serves all children with autism and their families, the core members have more power in the NGO, leading to “elite capture” and generating inequality. Elite capture refers to the phenomenon whereby elites form an interest alliance and jointly monopolize public resources (69). In CJ, a small number of core members directly or indirectly decide the allocation of internal resources. Elite members and other families with more intimate relationships through blood, geography, and occupation are more likely to have access to social inclusion activities. This excludes families with weak social networks. Many parents of children with autism who want to participate in these activities say that they have been cheated in the face of widespread unfair distribution.

In order to help my kid recover, we have participated in many activities from NGOs. I feel that these NGOs inside also have a circle, that is, there are close and distant relationships within them. Just look at CJ. When there are activities with no limit for the number of participants, my kid can participate. If it is a good activity with a limit of 20 or 30 participants, it will not be available for my kid. Only those inside the organization have the opportunity. (Interview with Yang, the parent of an autistic child, April 21, 2021)

#### 4.3.2. Small-scale crowdfunding-based NGOs for children with autism: dilemma of survival with a lack of funds

DK is jointly established and maintained by parents of children with autism, but compared with an elite-funding-oriented NGO, it faces a relatively severe shortage of funds which has created a series of difficulties, such as a lack of management personnel, a shortage of professional talent, and project sustainability difficulties. Although the leaders of DK have made great efforts to maintain the operation of the organization, in terms of practical effects, they cannot provide professional, effective, and continuous services, which makes it difficult for them to have a meaningful effect on social inclusion.

##### 4.3.2.1. Difficult to continue

Lack of funds leads to intermittent activities which fail to achieve the goal of long-term social inclusion. DK’s director, Lee, told us confidently that *“you can do things with lots of money, and you can also do things without money” (Interview with Lee, May 2, 2021)*, and she listed DK’s typical inclusion activities, such as collective life and art therapy. However, the reality is that many activities have been forced to terminate due to a lack of funding.

*To be honest, we have a lot of good ideas. But often just because we do not have enough support, we cannot do it... Our lack of funds is the biggest problem. Without money, there is no way to organize activities and hire professional teams. Now, we mainly charge for some of our activities and get help from some kind-hearted people. And some volunteers come to help us organize our activities. But there is still a big gap in our expectations. (Interview with Jane, May 8, 2021)*

##### 4.3.2.2. A vicious cycle

To make matters worse, inadequate funding creates a vicious circle, thus failing to improve the situation. DK has more than 220 members, but the economic situation of most of these families is average or even poor. The members are more conservative in their use of money, and most of them want to be free-riders. Deputy director Jane explained that the biggest problem with the DK is that,

When it comes to low-cost activities, people are very much motivated. When the cost of a project is high and everyone needs to contribute money to carry it out, parents are on the fence. (Interview with Jane, May 8, 2021)

This creates a development dilemma: Due to inadequate funds, it is difficult for DK to provide services in a long-term and stable manner as this would require more parents to invest funds. But parents are less willing to spend money because they do not see any prospect of the project. Under this vicious circle, NGOs such as DK passively reject truly good social inclusion projects. Ming, the principal of AH, the government-oriented NGO, is not satisfied with DK’s performance,

*DK is still where it started after all these years. It has not had any success in training any children with autism. (Interview with Ming, June 2, 2021)*

We must admit that every type of individual-financed NGO is looking for ways to improve its own ability to survive in order to achieve the social inclusion of children with autism. However, whether NGOs have good funds or poor funds, it is difficult to achieve the established goals under the current environment.

## 5. Conclusion and discussion

Using the case study method and an NGO classification approach, this study answers the question of why, in China, a large number of children with autism fail to achieve the goal of social inclusion through NGOs. NGOs for autistic children, as important direct welfare providers, have a very critical impact on the future development of children with autism. Since the 1990s, Chinese parents who have children with autism and caring people concerned about autism have set up NGOs to provide services for these children. In the course of their development, these NGOs have become divided according to the different funding sources they rely on. This paper categorizes them as government-oriented NGOs, foundation-supported NGOs, and individual-financed NGOs. Through extensive and in-depth field research, we found that the structural characteristics of sponsors and their interactions with recipients shape their different action logics.

Specifically, the actions of NGOs for children with autism in an asymmetric dependence relationship with sponsors must meet the requirements of their sponsors. Therefore, the respective characteristics of the three types of NGOs for children with autism in the service field need to be redefined and examined. First of all, government-oriented NGOs for children with autism rely on government purchasing services to establish close ties with the government, but at the same time, being seen as the spokespersons of the local DPFs and the performers of administrative tasks, they do not play the role of protecting children with autism. In the face of the institutional environment constraints of the complete decision-making power, the absolute bargaining power, and the strict assessment power of the local DPFs, government-oriented NGOs prefer to simply fulfill tasks, which lead to issues of age exclusion, service type exclusion, the reduction of service quality, and the replacement of service content. Second, foundation-supported NGOs for children with autism need to win trust and support from foundations through project competition. However, the continuous competition leads to their paying attention to the “quantity” of influence rather than “quality” – the pursuit of numbers of retweets and likes replaces the substantive goal of realizing the social inclusion of children with autism. This study also found that faced with financial temptation from several foundations, foundation-supported NGOs tend to select children with mild autism to meet their assessment goals, leading to a large number of children with severe autism being ignored. While these stronger NGOs become the winners in this competitive environment, less developed NGOs and the children with autism they serve are left out of the service system. Finally, our interviewees provided clear evidence that leaders and core members are exceptionally important in individual-financed NGOs. These NGOs can be divided into two categories on the basis of the different identities of their core team members: elite-funding-oriented NGOs and small-scale crowdfunding-based NGOs. For elite-funding-oriented NGOs, the large influx of funds easily leads to interest priority and elite capture, resulting in unequal access to services for all members and even acquiescence to some exclusive behaviors in the construction of an inclusion environment. For small-scale crowdfunding-based NGOs, the financial difficulties they face means that their activities are always intermittent and they are unable to implement the most ideal social inclusion projects; such unsatisfactory results create a vicious circle and ultimately fail to meet the social inclusion needs of children with autism.

An increasingly important topic in regard to the current service provision for children with autism in China is how different types of NGOs can better provide social inclusion services. We have mapped out a tripartite interaction between service funders (governments, foundations, or individual donors), providers (NGOs), and service users (children with autism and their parents) and defined the roles and functions of these three parties in the area of social service provision. But the experience of NGOs shows that in a state of resource dependence, they inevitably give priority to the requirements of funders rather than responding to the needs of children with autism and their families. How to establish a tripartite two-way supply and demand closed loop and establish a more equal interactive relationship should be the focus of social services for children with autism in the future. This not only requires NGOs for children with autism to redefine their own roles but also, more importantly, taking the rights of children with autism and their families’ needs fully into account.

From the perspective of NGO classification, this study found that different NGOs have different motivations for funding dependence, resulting in different exclusion of social inclusion services for autistic children. In China, the authoritarian government has had a huge impact on this result. In this study, the different interaction logics of funders and NGOs in specific environments should provide some reference for the provision of social services for children with autism in other countries in the future. At the same time, it must be admitted that this study has some limitations. First, only three common types of NGOs for autistic children were analyzed in this article. In fact, there are various types of NGOs for autistic children, which require a more comprehensive understanding. Second, there are few studies on relationship of autistic children with NGOs, making it difficult to obtain large amounts of information from previous studies. Third, this study is based on a qualitative field survey of one site, and so the representativeness and universality of its findings need to be further verified in future studies. Finally, we emphasize the potential replication of this study, but the applicability of its results to other countries needs to be demonstrated.

## Data availability statement

The raw data supporting the conclusions of this article will be made available by the authors, without undue reservation.

## Ethics statement

Ethical review and approval was not required for the study on human participants in accordance with the local legislation and institutional requirements. Written informed consent for participation was not required for this study in accordance with the national legislation and the institutional requirements.

## Author contributions

ZL: conceptualization, methodology, investigation, writing – original draft, writing –revised draft, writing – revised draft, administration, and funding acquisition. CQ: conceptualization, methodology, investigation, writing – original draft, and supervision. All authors contributed to the article and approved the submitted version.

## Funding

This study supported by the Humanities and Social Sciences Project of Shandong Province (J18RA009).

## Conflict of interest

The authors declare that the research was conducted in the absence of any commercial or financial relationships that could be construed as a potential conflict of interest.

## Publisher’s note

All claims expressed in this article are solely those of the authors and do not necessarily represent those of their affiliated organizations, or those of the publisher, the editors and the reviewers. Any product that may be evaluated in this article, or claim that may be made by its manufacturer, is not guaranteed or endorsed by the publisher.

## Footnotes

^1^Also known as the dual management examination and approval registration system, meaning that the establishment of NGOs needs to be approved by the competent business units and registration authorities. The system of direct registration was adopted in 2012, phasing out the “dual management system.”

^2^NGOs for autistic children cannot collect membership fees.

^3^Government purchasing of services is an effective way to provide social services. By market mechanism, the provision of some public services directly provided by the government is entrusted to qualified NGOs, with the government paying the fees according to the contract.

^4^The China Disabled Persons’ Federation, founded in March 1988, is a national organization for all persons in China who have diverse disabilities, with the guiding principles of representing, serving, and managing persons with disabilities. Local DPF have been established all over mainland China at the provincial, prefectural, county, and township levels, with the mission of promoting the full and equal participation of people with disabilities in society and of ensuring that people with disabilities share the material and cultural achievements of society.

^5^We found that there is a third type, namely, parent-buying NGOs for children with autism, but such organizations are rare. At the same time, parent-buying NGOs closely resembles the market: that is, charging low fees leads to survival difficulties, while charging high fees leads to market exclusion – consistent with previous literature. Thus, this paper will not go into details later.

^6^In China, social inclusion services for autistic children are provided through Project System, including the following links. The first is the project application. The NGOs shall formulate feasibility plans or application reports and submit them to the project sponsor. The second is the project approval. The project sponsor determines the funded project according to the application report and comprehensive consideration of other factors. The third is project implementation. NGOs provide corresponding social services according to project contracts. The fourth is the project assessment. In the process of project implementation and settlement, there will be multiple assessment at different phases from the project sponsor.

^7^In 2015, Tencent 99 Charity Day was launched by Tencent Charity Foundation, which is one of the most influential projects. Tencent Charity Foundation was founded in June 2007, which is the first national non-public Internet foundation. The foundation is committed to promoting the development of the public welfare industry through the Internet, especially the mobile Internet technology and services.
